# Cancer-Related Neuropathic Pain

**DOI:** 10.3390/cancers11030373

**Published:** 2019-03-16

**Authors:** Helen L. Edwards, Matthew R. Mulvey, Michael I. Bennett

**Affiliations:** Academic Unit of Palliative Care, Leeds Institute of Health Sciences, University of Leeds, Leeds LS2 9NL, UK; m.r.mulvey@leeds.ac.uk (M.R.M.); m.i.bennett@leeds.ac.uk (M.I.B.)

**Keywords:** neuropathic pain, cancer, pharmacotherapy

## Abstract

Neuropathic pain in cancer is common and debilitating. It is important to differentiate neuropathic pain from other cancer-related pains as it is associated with worse pain outcomes and requires different treatment strategies. This review summarises recent updates to pain classification, aetiology, pain assessment and current recommendations for treatment in patients with cancer-related neuropathic pain.

## 1. Definitions and Epidemiology

Neuropathic pain is defined as “pain caused by a lesion or disease affecting the somatosensory system” [1] and may lead to both loss of function and also increased pain sensitivity and spontaneous pain [2,3]. It is common in cancer, resulting from direct damage to the nervous system from a primary tumour or metastases, or from cancer treatment, such as chemotherapy [4]. Neuropathic pain is usually chronic, either persisting continuously or characterised by recurrent painful episodes [2].

Neuropathic pain is different from nociceptive pain, which is defined as “pain that arises from actual or threatened damage to non-neural tissue and is due to the activation of nociceptors” [5]. In nociceptive pain, the somatosensory nervous system is functionally normally, contrasting with abnormal function underlying neuropathic pain. It is estimated that 20% of cancer pain is purely neuropathic in origin [6]. However, when mixed neuropathic-nociceptive pain is included, approximately 40% of patients with cancer are affected by neuropathic pain [6].

Neuropathic cancer pain is associated with poor outcomes [7,8]. A study in 2012 found that neuropathic cancer pain was associated with more oncological treatment, greater analgesic requirements (including strong opioids and adjuvant analgesics) and reduced performance status than those with nociceptive pain [8]. Patients with neuropathic pain also reported worse physical, cognitive, and social functioning [8].

In recognition of the importance of identifying neuropathic pain, the latest International Association for the Study of Pain (IASP) classification of chronic pain for International Classification of Diseases (ICD-11) identifies specific codes for chronic neuropathic cancer pain (neuropathic pain caused by tumour), as well as neuropathic pain caused by treatments, such as chemotherapy or radiotherapy [4]. The IASP guidance emphasises the importance of a correct diagnosis of the pain in cancer so that tailored treatment (including analgesia, anticancer therapies, surgery and non-drug interventions) can optimise pain outcomes [9].

## 2. Aetiology

The aetiology of cancer-related neuropathic pain is diverse. Cancer-related neuropathic pain is conventionally subdivided into tumour-related and treatment-related pains. Not all pain in a cancer patient is cancer-related; pain from comorbid diseases is common, particularly in older patients [10]. About two-thirds of neuropathic pain in cancer patients is tumour-related (neuropathic cancer pain [NCP]), around 20% results from cancer treatment and 10–15% from comorbid diseases [6]. The proportion of pain caused by cancer treatment is higher in neuropathic pain compared with all types of cancer pain [6].

NCP may result from direct infiltration by the primary tumour or metastases into components of the peripheral or central nervous system. Examples of involvement of the peripheral nervous system include invasion of the brachial plexus by thoracic tumours, or invasion of the lumbosacral plexus by abdominal or pelvic tumours. A typical example of involvement of the central nervous system is spinal cord compression resulting from vertebral collapse due to bony metastatic disease.

Paraneoplastic neurological syndromes can also occur, resulting from remote effects of cancer mediated by the immune system [11]. They can affect the function of the nervous system at any level, including the central nervous system, peripheral nervous system, neuromuscular junction and muscle itself [11]. The most frequent paraneoplastic neurological syndromes are paraneoplastic cerebellar degeneration and sensory neuronopathy [11,12].

Cancer therapy-induced neuropathic pain can result as an unintended consequence (side effect) or complication from cancer treatments, including surgery, radiotherapy and chemotherapy. Surgical intervention may cause direct damage to peripheral nerves. Examples of interventions commonly associated with post-traumatic neuropathic pain syndromes include mastectomy and thoracotomy [4,10].

Radiation treatment can result chronic painful radiation-induced neuropathy. Delayed local damage to the nervous system in the field of radiotherapy is common [4], sometimes manifesting months or even years following treatment [10]. It is often progressive and irreversible [4]. The underlying mechanisms are not fully understood. It may result from nerve compression from radiation-induced fibrosis, but also from direct nerve and blood vessel injury from microvascular changes [13]. Brachial plexopathy following irradiation for lung or breast cancer is a classic example of radiation-induced neuropathy [14]. Other less common examples include painful lumbosacral plexopathy after pelvic radiotherapy [13,15] and axial neuropathy of the spinal cord following cervical radiotherapy [4].

Chemotherapy-induced peripheral neuropathy (CIPN) is a common, painful and dose-limiting side effect of chemotherapy [10]. It is generally dose-dependent [16]. Typically, symptoms begin in the first two months of treatment, worsen as treatment progresses and then stabilise soon after cessation. However, symptoms can persist long after treatment. A systematic review in 2014 found that CIPN affects 60% of patients 3 months after treatment, and 30% at 6 months or more [17]. Common neurotoxic chemotherapeutics include the taxanes (paclitaxel and docetaxel), platinum-based drugs (cisplatin and oxaplatin), vinca alkaloids (vincristine), thalidomide and proteasome inhibitors (bortezomib) [4,10,16]. CIPN most commonly results from direct neurotoxic effects of chemotherapy on dorsal root ganglion neurons or their axons, causing pain in a stocking-and-glove distribution, sensory loss and sensory ataxia [16]. The pain is often characterised by pricking or burning, or an “electric sensation” [4]. Autonomic, motor and occasionally cranial nerve involvement can less commonly occur [16].

## 3. Assessment

Clinically, neuropathic pain is a heterogeneous entity, comprising many and varied positive and negative symptoms. Positive symptoms include pain, both spontaneous and evoked. Spontaneous pain can be continuous or paroxysmal. Evoked pain can result from non-nociceptive stimuli (e.g. pressure from tight clothes or stroking) causing allodynia, or as an increased response to a nociceptive stimulus causing hyperalgesia [2,18]. Characteristics of neuropathic pain include shooting, sharp, stabbing, tingling, pricking, electric shocks and pins and needles [19,20]. Negative symptoms include decreased or loss of sensation to light touch and vibration (hypoesthesia) and pain (hypoalgesia) [21]. Autonomic features sometimes associated with neuropathic pain include mottled skin, sweating, redness or swelling [20].

Neuropathic pain can be difficult to identify, particularly in the context of cancer pain, where other processes, such as pre-existing neurological disease or muscle spasticity from disorders of the motor system, may confuse the clinical picture [22]. This has implications both in clinical and research settings. In clinical practice this may lead to under recognition, and therefore undertreatment, of neuropathic pain. In research, inadequate pain assessment can lead to heterogeneous sample populations and an increasing number of neuropathic pain studies failing to reach their primary efficacy end point [23,24], resulting in a poor evidence base for treatment recommendations. 

**Figure 1:**
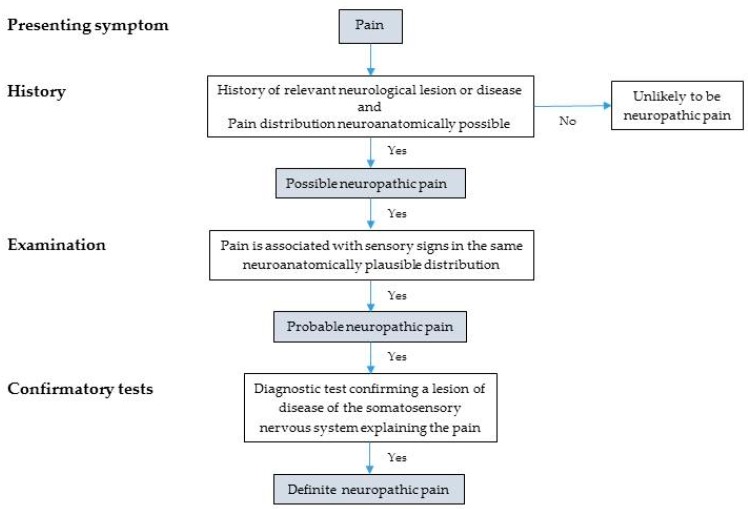
NeuPSIG upgraded system for neuropathic pain. Adapted from Finnerup et al. [22].

In an effort to address this, in 2016 the International Study for the Study of Pain (IASP) Special Interest Group on Neuropathic Pain (NeuPSIG) proposed an updated grading system for assessment of neuropathic pain, for use in both clinical and research settings (Figure 1). This system stratifies pain into possible, probable and definite neuropathic pain [22]. The assessment criteria include: (1) history of relevant neurological lesion or disease and neuroanatomically plausible distribution of pain; (2) pain associated with sensory signs in the same plausible neuroanatomical distribution; and (3) a diagnostic test confirming the presence of a lesion or disease of the somatosensory nervous system that explains the pain. Satisfying these three criteria in turn raises the likelihood of neuropathic pain from possible to probable to definite. The authors suggest that a “probable” diagnosis of neuropathic pain should be sufficient to initiate treatment for neuropathic pain. They also suggest that “definite” diagnoses will generally be limited to specialist contexts, and where treatment targeted at the underlying lesion or disease may be possible [22].

Diagnosis of neuropathic pain in cancer is no different from other clinical diagnoses, relying upon comprehensive clinical history, examination and diagnostic tests. The NeuPSIG grading system and other screening tools can be usefully applied in clinical practice to identify probable cases of neuropathic pain, with little deviation from normal practice. For example, the NeuPSIG grading system starts with clinical history. This should include sensory descriptors suggestive of a neuropathic mechanism (e.g., shooting, pricking, pins and needles, numbness and tingling) with a relevant neurological lesion, and distribution of these symptoms in the corresponding neuroanatomical distribution. Clinical examination for sensory changes may be a little more involved than in routine clinical practice, but is still feasible at the bedside. It involves assessment of touch, vibration, pinprick, cold and warmth using routinely available tools, such as a cotton bud, tuning fork, toothpick and teaspoons in warm and cool water. The grading system indicates that only one sensory abnormality needs to be present to satisfy this criterion. Lastly, many of the diagnostic tests that would support a definite diagnosis of neuropathic pain according the NeuPSIG are routinely performed anyway, such as computed tomography or magnetic resonance imaging. Other more specialist tests may include neurophysiological tests, such as nerve conduction velocity for example [22].

The NeuPSIG criteria have not yet been universally adopted as the reliability (inter-rater and test-retest) and applicability in clinical practice and research has not been established [23]. Other screening tools are available for use in clinical and research settings. The most commonly used neuropathic pain screening tools in research are the Leeds Assessment of Neuropathic Symptoms and Signs (LANSS) [20], the Douleur Neuropathique en 4 (DN4) [25] and painDETECT (PDQ) [26]. The LANSS comprises five sensory symptom items and two clinical examination items, DN4 comprises seven symptom items and three clinical examination items and painDETECT is a screening questionnaire comprising nine symptom items [27]. These tools were largely developed in patients without cancer and are biased towards detecting positive sensory abnormalities. NCP is unique in that pain is often experienced in the presence of sensory loss; therefore, it has been argued that existing screening tools may not perform adequately in cancer populations. A study in 2017 assessed performance of these screening tools in identifying neuropathic pain in patients with cancer [23]. They found that LANSS and DN4 screening tools could generally distinguish between neuropathic and not neuropathic pain in patients with cancer, although not with the same degree of accuracy as found in non-cancer populations. Nevertheless, these screening tools can be useful in identifying potential cases of neuropathic pain in cancer until more accurate tools are developed.

Another approach to pain assessment generally reserved for use in research settings is quantitative sensory testing (QST). QST is a detailed assessment of sensory function and provides information about the underlying neurobiological mechanisms of pain processing. A standardised QST protocol published by the German Neuropathic Pain Consortium (DFNS) in 2006 [28] has been widely accepted in clinical research. However this protocol has had limited impact on clinical practice as QST is labour intensive and requires highly trained operators with expensive equipment. Nevertheless, QST data from clinical trials indicate that sensory abnormalities are common in cancer patients with pain [29,30,31,32]. These data suggest that integrating simple bedside assessment of sensory function (as described in the NeuPSIG grading system) may improve the detection and diagnosis of neuropathic cancer pain. QST data have also demonstrated that the presence of pre-existing sensory deficits (particularly light touch) prior to chemotherapy are a contributing factor in the onset of painful CIPN [33,34].

## 4. Management

The evidence base for management of cancer-related neuropathic pain is limited. There is no clear consensus for first-line or step-wise treatment of cancer-related neuropathic pain [19,35], which can prove challenging for patients and clinicians alike. Guidelines for pharmacological and other management of neuropathic cancer pain have recently been published by the European Society for Medical Oncology (ESMO) [19]. Pharmacological treatment options include opioids, non-opioids and “adjuvant analgesics”, either alone or in combination. Adjuvant drugs are those traditionally used for a primary indication other than pain, but with analgesic properties under some circumstances [36]. These agents are often used first-line for neuropathic pain, so the term is something of a misnomer but is still often used [36,37]. Adjuvant agents used in neuropathic pain include tricyclic antidepressants (TCAs) and anticonvulsants. Steroids are another adjuvant drug which can be used in cases of nerve compression causing neuropathic pain. There are strong recommendations against the use of levetiracetam and mexiletine [19].

Gabapentin, pregabalin, duloxetine and TCAs (doses of ≤75 mg/day) are recommended in the ESMO guidance as single agents for first-line treatment of neuropathic pain in cancer. This recommendation has been extrapolated from systematic reviews in non-cancer related neuropathic pain. For example, a 2015 systematic review and meta-analysis of pharmacotherapy for neuropathic pain in adults found that the numbers needed to treat were 3.6 for TCAs, 6.4 for serotonin-noradrenaline reuptake inhibitors (mainly duloxetine), 7.7 for pregabalin and 7.2 for gabapentin [9]. They also found that the efficacy of drug treatments was not generally dependent on the underlying cause of neuropathic pain, which perhaps supports extrapolation of the findings to cancer-related neuropathic pain.

Some studies have examined the role of adjuvants specifically in neuropathic pain in cancer but the data are less conclusive. A systematic review in 2017 examined the role of adjuvants in treating cancer pain, both purely neuropathic and mixed cancer pain [36]. They included gabapentin, pregabalin, amitriptyline and venlafaxine, and found low quality evidence for these agents in reducing pain intensity in patients with cancer pain.

Cancer-related neuropathic pain can also be treated using opioids or non-opioids, in combination with TCAs or anticonvulsants where required. The evidence for this is not conclusive, however. A systematic review in 2011 examined the effect of adding antidepressant or antiepileptic drugs to opioids for cancer pain that was largely caused by neuropathic mechanisms. Clinical and methodological heterogeneity precluded meta-analysis but a narrative analysis found that the addition of these adjuvants to existing opioid analgesia provided a modest improvement in pain intensity after 4 to 8 days, but unlikely more than 1 point on a 0–10 numerical rating scale, and with increased risk of adverse events [38]. The evidence of benefit was strongest for gabapentin. An update to this review was able to perform meta-analysis of trials that examined the effect of adding antidepressants or antiepileptics to opioids, comparing cancer pain outcomes between opioid/adjuvant combinations with opioid monotherapy [39]. This showed no additional improvement in tumour-related pain relief from adding gabapentinoids to opioids, and an increase in adverse events. However, again the quality of the evidence was low and benefit for patients with definite neuropathic cancer pain could not be excluded. The authors concluded that there remains uncertainty regarding the risk-benefit trade off from combining adjuvants and opioids for treatment of tumour-related cancer pain. The ESMO guidance advocates careful dosing and monitoring of side effects if using adjuvants in combination with opioids for neuropathic pain.

Other treatment options discussed in the ESMO guidance include ketamine and interventional treatments. Ketamine is an N-methyl-D-aspartate (NMDA) antagonist which has been used to treat refractory cancer pain, including neuropathic pain, but current evidence is insufficient to determine the benefits and harms associated with this treatment [40]. Interventional treatments of neuropathic pain in cancer are not recommended in the ESMO guidelines as the evidence is weak or inconclusive, unless used for neuropathic pain syndromes unrelated to cancer [19].

Another evolving approach to analgesia, including in cancer-related neuropathic pain, is the concept of mechanism-based pain management [41,42]. This involves assessment of pain aetiology (e.g. inflammatory or neuropathic), location of pain-generating mechanism (e.g., peripheral or central sensitisation) and corresponding molecular targets (e.g., nerve growth factors) [42]. This is an exciting area of research with myriad potential new analgesic targets, but at present there are no licensed targeted treatments and no clinical studies demonstrating efficacy in cancer patients.

Non-pharmacological interventions are also important in multimodal management of neuropathic cancer pain, as in all forms of cancer pain. It is well-recognised that inadequate patient education can provide a barrier to effective pain management in cancer [43,44]. There is also increasing evidence of the importance and effectiveness of patient education in reducing pain intensity and pain interference in cancer patients [45,46]. Self-management interventions for people with cancer pain are one such example. These aim to increase patients’ knowledge, skills and confidence to manage their condition [47], and importantly, to become active in their own management [48].

## 5. Future Research

Considerable future research challenges for neuropathic pain in cancer lie ahead. Priority areas for research include cancer pain assessment, pain neurobiology and targeted therapies. Comprehensive and accurate cancer pain assessment is essential, not only to differentiate neuropathic from nociceptive pain, but also in more precisely profiling the characteristics of neuropathic pain. It is increasingly recognised that neuropathic pain actually comprises a range of different subgroups or so-called sensory profiles [49,50]. These may result from different neurobiological mechanisms, and so may respond differently to treatments tailored to these. Therefore, careful pain assessment can indicate pain mechanisms and guide targeted therapy, improving pain outcomes but also improving the evidence base for treatment of this particularly challenging symptom.
